# Cognition and interpersonal coordination of patients with schizophrenia who have sports habits

**DOI:** 10.1371/journal.pone.0241863

**Published:** 2020-11-09

**Authors:** Keisuke Fujii, Yujiro Yoshihara, Yukiko Matsumoto, Keima Tose, Hideaki Takeuchi, Masanori Isobe, Hiroto Mizuta, Daisuke Maniwa, Takehiko Okamura, Toshiya Murai, Yoshinobu Kawahara, Hidehiko Takahashi

**Affiliations:** 1 Graduate School of Informatics, Nagoya University, Nagoya, Japan; 2 Center for Advanced Intelligence Project, RIKEN, Tokyo, Japan; 3 Department of Psychiatry, Graduate School of Medicine, Kyoto University, Kyoto, Japan; 4 Department of Psychiatry and Behavioral Sciences, Graduate School of Medical and Dental Sciences, Tokyo Medical and Dental University, Kyoto, Japan; 5 Takatsuki Sports Club for Mental Illness, Takatsuki, Japan; 6 Shin-Abuyama Hospital, Osaka, Japan; 7 Institute of Mathematics for Industry, Kyushu University, Fukuoka, Japan; Chiba Daigaku, JAPAN

## Abstract

Team sports activities are effective for improving the negative symptoms and cognitive functions in patients with schizophrenia. However, the interpersonal coordination during the sports and visual cognition of patients with schizophrenia who have team sports habits are unknown. The main objectives of this study were to test two hypotheses: first, patients with schizophrenia perform the skill requiring ball passing and receiving worse than healthy controls; and second, the patients will be impaired in these functionings in accordance with the previous studies regarding schizophrenia in general. Twelve patients with schizophrenia and 15 healthy controls, who had habits in football, participated in this study. The participants performed three conventional cognitive tests and a 3-vs-1 ball possession task to evaluate their interpersonal coordination. The results showed that in the 3-vs-1 possession task, the displacement in the pass angle for the patients was significantly smaller than that for the control. The recall in the complex figure test, the performance in the trail making test, and that in the five-choice reaction task for the patients were worse than those for the control. Moreover, we found the significant partial correlations in the patients between the extradimensional shift error and the pass angle as well as between the time in the trail making test and the displacement in the pass angle, whereas there was no significant correlation in the control group. This study clarified the impaired interpersonal coordination during team sports and the visual cognition of patients with schizophrenia who have team sports habits.

## Introduction

Exercise and physical activity have positive effects on various mental disorders and produce therapeutic benefits [1–3] such as in emotions (e.g., depression), cognitive functions (e.g., attention and memory), sociality, and stress coping. Patients with mental disorders who regularly exercise have been found to have a higher health-related quality of life [4]. Systematic reviews have reported that exercise intervention in patients with mental disorders improved the negative symptoms and cognitive functions [5] such as working memory, attention, and alertness [6].

Among the various types of exercises, team sports such as football and basketball require social interaction with visual cognition and executive functioning, in addition to the individual exercise such as running and dribbling. Interpersonal coordination, i.e., adjusting an individual’s movements while reading others’ intentions and predicting their movements in such team sports [7, 8], are considered to be related with such as social cognition (e.g., biological motion perception) [9]. Several studies reviewed by [10] have reported the positive effects of football-based interventions (e.g., self-reported quality of life [11] mainly through interviews as a longitudinal study), especially in patients with schizophrenia [11, 12].

However, cognitive characteristics in patients with schizophrenia who have team sports habits and their interpersonal coordination during sports are unknown (even as a cross-sectional study). In general, patients with schizophrenia have impairments in several levels of cognition including perception, attention [13], and social functioning including communicating with others, maintaining employment, and functioning in the community [14]. Then, as a first step, we investigate cognition and interpersonal coordination during team sports by comparing the patients and controls with team sports habits. The previous studies investigated interpersonal coordination using the hand-held pendulum synchronization tasks [15, 16] and demonstrated that intentional social motor coordination was impaired in patients with schizophrenia. In contrast, to the best of our knowledge, this is the first study to investigate cognition and interpersonal coordination during team sports (i.e., requiring more complex cognitive and motor interaction) among patients with mental disorders.

In team sports sciences, interpersonal coordination in athletes has been intensively investigated in small-sided [17–19] and actual games [8, 20, 21]. However, the quantitative evaluation of such interpersonal coordination during sports activities in patients with mental disorders has been unknown. We firstly hypothesized that patients with schizophrenia would perform skills requiring ball passing and receiving a ball worse than healthy controls, because patients with schizophrenia have impaired cognitive functions [22]. To test the hypothesis, we employed a 3-vs-1 ball possession task in football [17, 18], which includes the fundamental passing and receiving skills.

From the viewpoint of psychiatry, generally in patients with schizophrenia, visual cognitive and executive functioning were impaired (e.g., [23–25]), which may be critical in the 3-vs-1 task. However, it remains unknown what cognitive characteristics in patients with schizophrenia who have team sports habits are similar to or worse than those in a control group. Here, we assessed their visual cognitive and executive functioning by employing cognitive tests described below. We secondly hypothesized that the patients will be impaired in these functionings according to the previous studies regarding schizophrenia in general (e.g., [23–25]).

The main purpose of this study was to test these two hypotheses. This study was exploratory in nature because it was the first study to investigate these functionings in patients with schizophrenia who have team sports habits. Moreover, we investigated the relationship among the performances in conventional cognitive tasks and motor tasks with the interpersonal coordination in the patients and the control group. This study contributed to clarifying the cognitive performances and the interpersonal coordination during team sports of patients with schizophrenia who have team sports habits.

## Materials and methods

### Participants

Twelve male patients with schizophrenia and 15 male normal controls, who had played in football, participated in this study. We recruited participants through the relations with some authors. All patients were home-living patients. Six of the twelve patients were working in employment for the handicapped, and the remaining six patients were visiting a day-care center. Any obvious abnormal movement of arms, legs, and posture like symptoms in Parkinsonism was not observed in the patients. For healthy controls, we recruited people with similar exercise levels to the patients in the same area. The patients were diagnosed with the patient edition of the Structured Clinical Interview for DSM-IV Axis I Disorders (SCID). We asked all participants to provide their age, height, weight (subsequently used to compute body mass index (BMI)), football experiences, and exercise during the last year. Football experiences consist of the experiences in total, those until 22 years old, and recent monthly experiences because there is a large variation in the ages of the participants. The second is the common football experience when young (as most Japanese people play football as students). The last is the common recent football experience. Their predicted IQ was measured using the Japanese version of the National Adult Reading Test (JART) short form, which is considered to reflect the premorbid IQ of patients/persons with schizophrenia [26]. For patients with schizophrenia, the Positive and Negative Syndrome Scale (PANSS) [27] was used to assess the severity of clinical symptoms with three subscales: positive, negative, and general psychopathology. Moreover, we used GAF (Global Assessment of Functioning) [28], of which ratings are positively associated with concurrent ratings of symptoms and social functioning.

This study was approved by the Committee on Medical Ethics of Kyoto University (R1667) and RIKEN (Kobe1 2018–10) and was conducted in accordance with the Code of Ethics of the World Medical Association. After a complete description of the study, we obtained written informed consent from all the participants. The participants performed the 3-vs-1 ball possession task, slalom test, and cognitive tasks. These tasks were selected based on our above hypotheses.

The reason why we recruited limited numbers of these participants (12 patients and 15 controls) is twofold: one is a scarcity of patients with schizophrenia who have football experiences and habits. Another is the difficulty in aggregating such people at the same place and time to play such as a 3-vs-1 task. For example, in sports science, studies in elite athletes have similar conditions [17]. In this field, the number of participants is not small at all and we believe that this study would be valuable as such a special case of patients with schizophrenia maintaining football habits, whereas we need to be careful in the interpretation of these results.

### The 3-vs-1 ball possession task

We employed a 3-vs-1 ball possession task because this basic and minimal setting includes the ball-passing and ball-receiving movements while predicting others’ motions [17, 18]. We asked the three attackers to keep possession of the ball from the defender and pass the ball to other attackers as much as possible within a 6-m square, and the defender was expected to make interceptions whenever possible. We defined a passer as the attacker with the ball, and potential receivers as the remaining two attackers without the ball (the receiver means the attacker who receives the ball). The patient and control group were divided into eight and 10 subgroups (or trials) of four players, respectively, to play an offensive role two or three times (three offensive roles in a subgroup were grouped for each patient and control group). They played the game for 60 s in each trial. For the control of the experimental condition, the participants in the control group played a defensive role once or twice.

Three-dimensional coordinates of the landmark points were acquired using a 3D optical motion capture system with 16 cameras (eight Prime 41 and W17 cameras, OptiTrack, NaturalPoint, USA) operating at 120 Hz. Reflective markers were placed at the top of each participant’s head. We also used the futsal ball pasted reflective sheet. All raw coordinate data points were smoothed using a second-order Butterworth low-pass digital filter (6 Hz).

**Fig 1:**
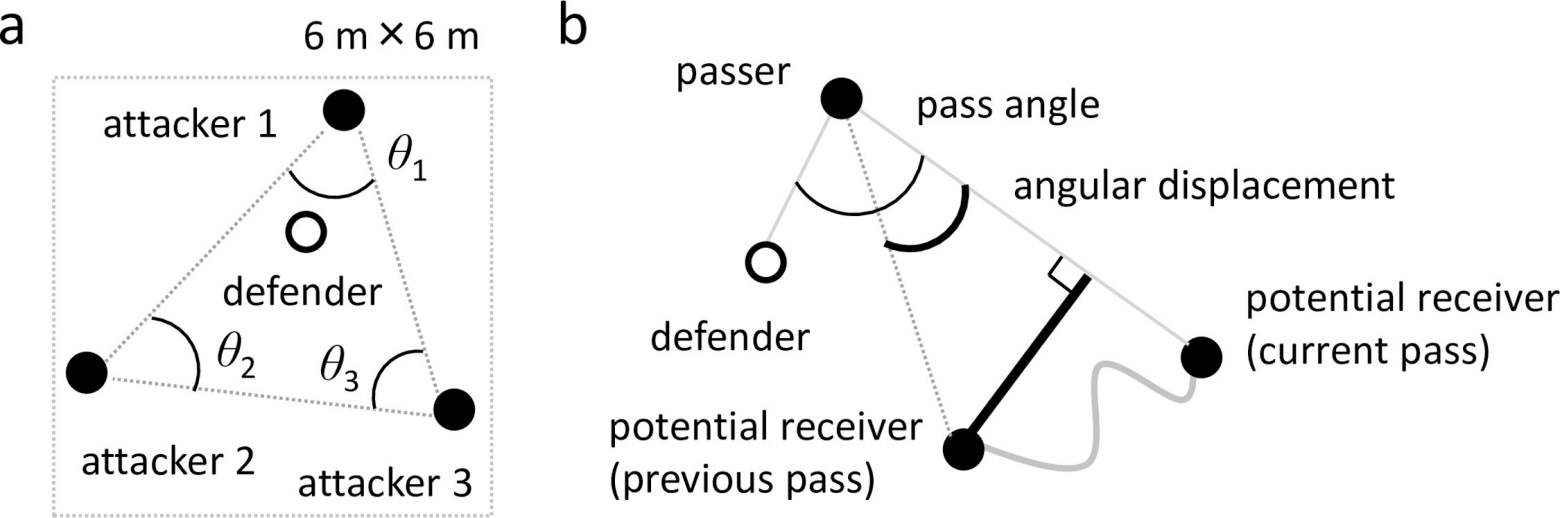
Diagram of 3-vs-1 variables. (a) The inner angles of the three attackers’ triangle. (b) The pass angles (thin arc) are defined as the angles between the vector from the passer to the potential receivers and the vector from the passer and the defender. We analyzed pass angles at the passer’s ball release. The angular displacement (thick arc) is defined as the difference in the above angle from the previous pass to the current pass (both ball releases). The additional distance is defined in the same time interval by subtracting the shortest distance (to obtain the same angular displacement; black thick line in Fig 1B) from the actual moving distance (gray curve in Fig 1B).

First, we evaluated the group coordination in the previous studies [17, 18] using three inner angles of the three attackers triangle (Fig 1A). Skilled athletes showed a higher spatiotemporal symmetry [17], which were quantitatively evaluated from the circular distribution of the phase plane in the three inner angle space of the attacker’s triangle. For quantitative evaluation, the previous study [18] evaluated the simulation model using the width of the (quasi-normal) distribution of the skilled athletes; thus we used the standard deviation (SD) of the three angles. It should be noted that the angles would not reflect the individual performance because the angle is influenced by the movements of three people. Furthermore, the previous papers evaluating the group coordination ignored the roles such as passer and receiver, by considering three attackers as homogeneous coupled oscillators. The main purpose of this study was to evaluate individual performances; thus, we evaluated the following variables.

Second, we computed the four variables for evaluating individual performances including interpersonal coordination: pass-miss rate, pass angle, angular displacement, and additional distance (Fig 1B). The pass-miss rate is a ratio of the number of unsuccessful passes to the total pass opportunities to evaluate the individual passing ability. We included all passing action to count the pass-miss rate, but we excluded unsuccessful passes and passes immediately after the pass-miss for computing the following variables for the analysis of the potential receivers. The pass angles are defined as the angles between the vector from the passer to the potential receivers and the vector from the passer and the defender (Fig 1B thin arc), which are called passing vector angles in the previous study [19]. We analyzed pass angles at the passer’s ball release, which is statistically related to the pass success [19] to evaluate the outcome of movements according to others’ movements (the passer and defender). The angular displacement is defined as the difference in the pass angle from the previous pass to the current pass (both ball releases: Fig 1B thick arc) to evaluate the movement process according to other’s movements. The additional distance is defined in the same time interval by subtracting the shortest distance (to obtain the same angular displacement; black thick line in Fig 1B) from the actual moving distance (gray curve in Fig 1B). This distance was used to evaluate the ineffective movement to receive the pass. The last three indicators are related to interpersonal coordination, because they should reflect the relationships with other participants.

### Slalom test

We used a slalom dribble test to evaluate an individual football skill such as dribbling other than the interpersonal coordination such as passing. The test was chosen because of the high test-retest reliability, and good ecological and construct validity [29]. The test assesses total body movement, requiring participants to dribble around a set obstacle course as quickly as possible. At the starter’s command, participants dribble the ball from behind the start line to the right of the first cone and then alternately around the outside of the remaining five cones in a zigzag path (31.72 m in total). Participants stop and leave the ball at the sixth cone before traveling in a straight line across the finish line (9.15 m). The total time was measured and recorded using photocells timing gates (Racetime2, Microgate). The participants were required to perform the slalom dribble twice, with the mean of both times used as the test score.

### Cognitive tasks

We employed three cognitive tasks: Rey-Osterrieth Complex Figure test (ROCFT), Trail Making Test A and B (TMT), and Cambridge Neuropsychological Test Automated Battery (CANTAB). These are related to visual cognitive and executive functioning and behavior, which are considered to be impaired in patients with schizophrenia and are critical in a 3-vs-1 possession game. ROCFT assesses visual memory, visual organization, and visuospatial abilities [30, 31]. The reproduction scores in immediate, 3-min, and 30-min delayed reproduction were investigated.

We used TMT to assess cognitive and motor abilities (e.g., [32]) by the amount of time required to complete the task to draw lines sequentially connecting the target (A: only numbers; B: alternately numbers and letters). TMT A involves attention, visual search, motor functioning, speed of processing, and working memory and TMT B also measures executive functioning [33].

CANTAB assesses cognitive and executive functioning and behavior. Among the 23 subtasks comprising CANTAB, the RTI (reaction time), IED (intra-extra dimensional set shift), and SWM (spatial working memory) were selected, because these require visuospatial cognition with time constraints. RTI [34] is a test of simple and five-choice reaction time. The outcome measures are simple and five-choice reaction and movement times. IED [35] tests cognitive flexibility, analogous to the Wisconsin Card Sorting Test, which has multiple stages segregating cognitive processes that assess rule learning, rule reversal, and attentional set-shifting. Outcome measures are (adjusted and normal) total errors and extradimensional shift (EDS) errors. SWM [36] is a self-ordered search task based on foraging behavior and assesses working memory and strategy use. The outcome measures are between- and within-search errors, as well as strategy. The high strategy score indicates that a participant found tokens in a more effective way.

### Statistical analyses

To compare the variables between groups, the Mann-Whitney U-test was used because most of the data often did not follow normal distributions using the Lilliefors test. For the same reason, we used Spearman’s rank correlation coefficient between the variables in the 3-vs-1 and cognitive tasks. To eliminate the effect of the related demographic and clinical profiles (i.e., controlling variables), we computed Spearman’s partial rank correlation coefficient. Since the input variables of the partial correlation can be completely explained by the controlling variables if all of the demographic and clinical profiles are used as controlling variables, it is necessary to select related demographic and clinical profiles. Consequently, in this study, we selected the recent monthly football experience and the duration of the illness. This is because we considered that the recent sports habits of non-elite athletes are more important than the experience, and longer duration of the illnesses associated with subtle motor and sensory neurological abnormalities in patients with schizophrenia [37]. The effect size was estimated using *r* = *z*/sqrt(*N*) for Mann-Whitney U-test, where *z* is *z*-statistic estimated by the Matlab function *ranksum* and *N* is the number of samples. For all the statistical calculations, *p* < 0.05 was considered as significant. All statistical analyses were performed using the MATLAB 2016a Statistics and Machine Learning Toolbox.

## Results

### Demographic and clinical profiles

The demographic data and clinical measures are shown in Table 1. The height, weight, BMI, predicted IQ, and football experience until 22 years old were matched between groups (*p* > 0.05). The total football experience of the control was significantly higher than that of the patients (*p* = 0.037, *r* = −0.40), but the age, exercise for the last year, and recent monthly football experiences for the patients were significantly higher than those in the control (*p* < 0.042, *r* > 0.39). Compared with the scores in more patients with schizophrenia (101 patients) of the previous study [27], the PANSS positive (14 ± 4.39) and negative (16.17 ± 6.53) in this study were smaller or similar compared to those in the previous one (18.20 ± 6.08 and 20.01 ± 6.17, respectively), suggesting that the patients in this study would be moderate in PANSS positive and negative compared to general patients with schizophrenia. GAF score for the patients with schizophrenia in this study was 55 ± 12.43, which was a similar level to the previous work (60.7 ± 14.22) in a larger sample size (216 patients) [38].

**Table 1:** Demographic and clinical data.

	Control	(N = 15)	Patients	(N = 12)	Statistics		
	Mean	SD	Mean	SD	p	r	
Age	32.87	2.32	39.25	2.27	0.022	0.44	*
Height	171.11	1.08	171.4	1.43	0.922	0.02	
Weight	69.1	2.6	73.59	3.59	0.204	0.24	
BMI	23.57	0.79	25.02	1.15	0.196	0.25	
Football experience in total (yr.)	13.4	1.87	8.25	1.14	0.037	-0.4	*
—until 22 yr. old (hr.)	3596	797.95	2262.5	735.34	0.187	-0.25	
Exercise for the last year (hr.)	268.47	61.01	411.25	76.62	0.04	0.39	*
Recent football per month (hr.)	3.73	1.01	7.67	1.89	0.041	0.39	*
Medication (mg/day, haloperidol equivalent)			388.58	159.48			
Duration of illness (yr.)			20.58	10.71			
Predicted IQ	107.67	1.4	104	3.16	0.462	-0.14	
PANSS positive			14	4.39			
—negative			16.17	6.53			
—general			29.42	7.43			
—total			59.58	15.73			
GAF			55	12.43			

* p < 0.05

### Performance in motor tasks between groups

**Fig 2:**
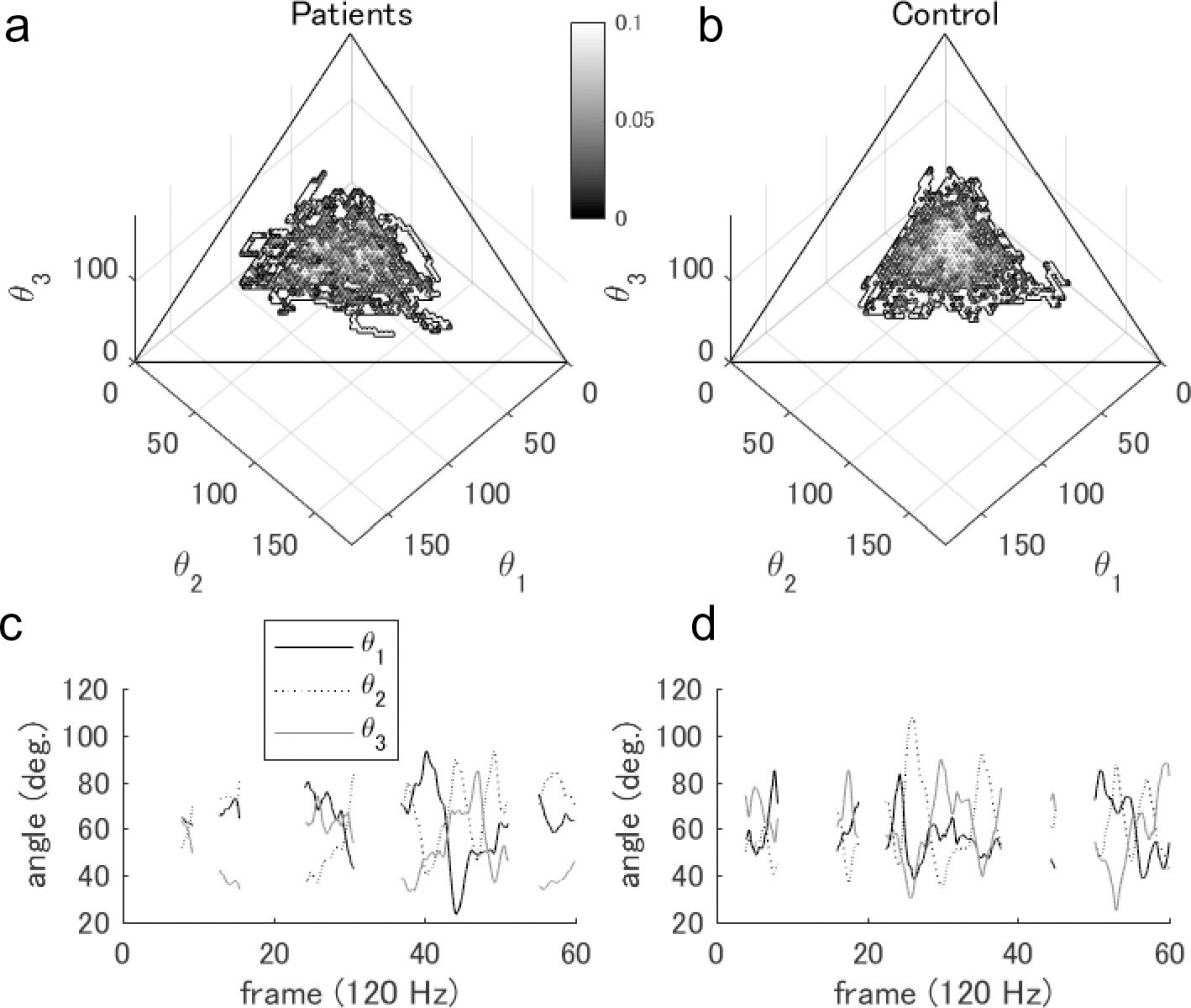
Comparison of group coordination between two groups. Grayscale contour plots in (a) the patients and (b) the control group indicate the normalized frequency in a three-angle space. The three angles indicate inner angles in the triangle of three attackers (see in Materials and Methods); thus, the plot was on the (two-dimensional) phase plane. The typical time series of the three angles in (c) the patients and (d) the control group are shown.

First, we demonstrated the differences in group coordination during the 3-vs-1 task. For the contour plots in the phase plane, the patient group in Fig 2A shows a slightly wider distribution than the control group in Fig 2B. Statistically, the SD of the angles for the patients (16.92 ± 0.93 deg.) was not significantly larger than that for the control (15.45 ± 1.14 deg.; *p* = 0.143, *r* = 0.35). Typical examples of three angles are shown in Fig 2C and 2D. These distributions in both groups visually seemed to be intermediate between the skilled athletes (smaller and circular distribution) and novices (wider and triangle distribution) in the previous study [17].

Next, individual performances in motor tasks are indicated in Table 2 (lower). The angular displacement in patients with schizophrenia was significantly smaller than that in the control (*p* = 0.043, *r* = −0.39). Meanwhile, there were no significant differences in the slalom test, pass-miss rate, pass angle, and additional distances (*p* > 0.05).

**Table 2:** Performances in cognitive tasks and motor tasks between groups.

	Control	(N = 15)	Patients	(N = 12)	Statistics		
	Mean	SD	Mean	SD	*p*	*r*	
Cognitive tests							
ROCFT immediately after	32	1	30.83	1.2	0.433	-0.15	
—3 min after	20.67	1.73	12.08	1.43	0.002	-0.6	*
—30 min after	20	1.63	12.13	1.61	0.005	-0.54	*
CANTAB RTI mean simple reaction time	267.88	8.38	291.75	15.64	0.252	0.22	
—mean simple movement time	418.87	22.05	469.79	46.39	0.575	0.11	
—five-choice movement time	284.29	7.62	318.71	13.56	0.022	0.44	*
—five-choice reaction time	282.53	8.27	312.38	13.4	0.048	0.38	*
CANTAB IED Total errors (adjusted)	19.53	5.3	26	6.11	0.129	0.29	
—EDS errors	7.20	2.18	10.33	3.34	0.431	0.15	
—total errors	14.53	2.74	19.75	3.06	0.129	0.29	
CANTAB SWM between search error	10.13	2.83	20.33	4.52	0.053	0.37	
—strategy	26.27	1.54	29	1.87	0.339	0.18	
—total error	10.6	3.05	20.75	4.56	0.06	0.36	
TMT A	61.47	2.2	89.58	6.86	0	0.7	*
—B	70.8	4.85	103.92	8.36	0.003	0.57	*
Motor tasks							
slalom	20.95	0.86	23.73	1.21	0.054	0.37	
3vs1 pass-miss rate	0.14	0.02	0.16	0.03	0.769	0.06	
—pass angle	36.25	0.97	35.69	0.93	0.643	-0.09	
—angular displacement	6.19	0.75	4.26	0.79	0.043	-0.39	*
—additional distance	0.60	0.04	0.49	0.03	0.092	-0.32	

* *p* < 0.05

### Performance in cognitive tasks between groups

The results for the cognitive tasks are presented in Table 2 (upper). The reproduction scores of ROCFT after 3-min and 30-min in patients with schizophrenia were significantly smaller than those in the controls (*p* < 0.006, *r* < −0.53). The scores of TMT (A and B) in the patients were significantly smaller than those in the control (*p* < 0.004, *r* < −0.56). In RTI of CANTAB, the five-choice reaction time and movement time in the patients were longer than those in the controls (*p* < 0.049, *r* > 0.37). All of these cognitive characteristics in the patients were worse than those in the control. Other characteristics, i.e., ROCFT immediate reproduction score, the performances in CANTAB RTI simple reaction task, IED, and SWM, were not significantly different between the two groups (*p* > 0.05).

### Correlation between 3-vs-1 variables and other variables

The results are presented in Table 3. Regarding cognitive tests, EDS error in CANTAB IED and TMT A were negatively correlated with the pass angle (*ρ* = −0.62, *p* = 0.031 and *ρ* = −0.64, *p* = 0.026) in patients with schizophrenia. For other characteristics, especially in all variables for the control group, there were no significant correlations between the interpersonal coordination and cognitive variables (*p* > 0.05).

**Table 3:** Correlations between variables in 3-vs-1 and cognitive tasks.

	Pass-miss rate		Pass angle		Angular displacement		Additional distance	
	Control	Patient	Control	Patient	Control	Patient	Control	Patient
Cognitive tests								
ROCFT immediately after	-0.01	0.3	0.15	-0.35	-0.2	-0.27	-0.19	-0.27
—3 min after	-0.16	0.02	0.13	-0.54	0.03	-0.26	0.04	-0.52
—30 min after	-0.06	0.18	0.26	-0.57	-0.25	-0.29	0.04	-0.34
CANTAB RTI mean simple reaction time	-0.01	-0.18	-0.33	-0.26	0.2	0.17	-0.25	-0.35
—mean simple movement time	-0.63	0.19	0.16	-0.19	0.2	0.14	-0.04	0.15
—five-choice movement time	-0.02	-0.12	-0.22	0.17	0.16	0.42	0.01	-0.09
—five-choice reaction time	0.03	-0.2	-0.16	-0.23	0.33	0.09	-0.18	-0.4
CANTAB IED Total errors (adjusted)	-0.07	0.02	-0.22	-0.51	-0.34	-0.18	-0.36	0.01
—EDS errors	-0.22	0.01	-0.15	-0.62	-0.36	0.09	-0.36	-0.07
—total errors	-0.07	0.02	-0.22	-0.51	-0.34	-0.18	-0.36	0.01
CANTAB SWM between search error	0.09	-0.07	0.36	-0.4	-0.34	-0.15	-0.18	0.18
—strategy	0.4	-0.06	0.25	-0.47	0.26	-0.16	-0.33	0.03
—total error	0.09	-0.07	0.36	-0.4	-0.34	-0.15	-0.18	0.18
TMT A	-0.11	0.01	0.19	-0.64	-0.14	0.3	-0.16	-0.06
—B	0.44	-0.11	-0.25	-0.27	-0.48	0.52	0.2	0.06
Profiles								
Football experience in total (yr.)	-0.04	-0.13	-0.43	-0.15	-0.22	0.15	-0.17	-0.25
—until 22 yr. old (hr.)	0.23	0.07	0.45	-0.31	0	0.15	0.13	-0.38
Exercise for the last year (hr.)	0.12	-0.56	-0.47	-0.09	0.32	-0.07	-0.07	-0.6
Recent football per month (hr.)	-0.04	-0.02	-0.44	0.23	-0.01	0.14	0.22	-0.08
Medication (mg/day, haloperidol equivalent)		0.58		-0.37		0.03		0.53
Duration of illness (yr.)		0		-0.17		0		0.24
GAF		-0.15		0.42		-0.03		-0.06
PANSS positive		0.14		-0.35		0.09		-0.47
—negative		-0.17		0.07		0.25		0.19
—general		-0.31		0.25		0.09		-0.26
—total		-0.15		0.09		0.24		-0.08

a: *p* < 0.05 in patient group

Next, we performed the partial correlation in the patients (the reason for selecting the controlling variable is described in Materials and Methods). There was a significant negative partial correlation between the pass angle and EDS error in CANTAB IED (*ρ* = −0.64, *p* = 0.046). Regarding angular displacement, there was a significant positive partial correlation with TMT B (*ρ* = 0.74, *p* = 0.014).

Additionally, we computed the correlations of the 3-vs-1 task performances with some demographic and clinical profiles including exercises, PANSS, duration of illnesses, and amount of medication. Regarding exercises, there were no significant correlations (*p* > 0.05) except for the correlation between additional distance and exercise for the last year (*p* = 0.039, *r* = −0.60). For the remaining clinical profiles, there were no significant correlations (*p* > 0.05) except for the correlation between pass miss rate and the amount of medication (*p* = 0.047, *r* = 0.58). Note that we mainly discussed the partial correlation results in the Discussion.

## Discussion

This is the first study to demonstrate interpersonal coordination in a 3-vs-1 possession task and visual cognitive tests in patients with schizophrenia and control groups. The first main result was that in the 3-vs-1 possession task, the angular displacement for the patients was significantly smaller than that for the control (discussed in the second paragraph). The second main result was that the recall in ROCFT, the performance in TMT, and that in the five-choice reaction task in CANTAB RTI for the patients were worse than those for the control (the third paragraph). Moreover, we found the significant partial correlations in the patients between the extradimensional shift error and the pass angle and between the time in the trail making test and the displacement in the pass angle, whereas there was no significant correlation in the control group (the fourth paragraph). This study clarified the impaired interpersonal coordination during team sports and the visual cognition of patients with schizophrenia who have team sports habits. However, since the sample size of our study was small, we need to be careful in the interpretation of these results.

In the 3-vs-1 ball possession task, the interpersonal coordination performance represented by the angular displacement in the patients was significantly lower than those in the controls. The displacement of the pass angle would be a critical variable in the task, which is independent of individual motor skills (e.g., slalom test performance). However, it should be noted that it is not always correct to increase the pass angle, because more expert athletes move dynamically to keep an equilateral triangle [17, 18] in the 3-vs-1 task. Meanwhile, the result revealing no significant differences in pass-miss, pass angle, and additional distance between the two groups did not support our hypothesis. The demographic profiles in Table 1 may affect the performances as a result. For example, significant results regarding the fitness and skills for patients (exercise for the last year and recent football experience) may have a potential not to make differences between the two group, but the significant results for the control group (football experience in total and age) and motor impairment in patients with schizophrenia [22] may have a potential to make the difference. We believe that we can discuss the results if considering the demographic profiles.

In the cognitive tests, the smaller reproduction scores of ROCFT after 3-min and 30-min (meta-analysis [23]) and smaller TMT scores in patients with schizophrenia (meta-analysis [24]) agreed with the results of the previous studies. In addition, these cognitive functions may not be improved by football habits. In contrast, CANTAB IED (review [39]) and SWM performances [25, 40] did not differ between the groups in this study, but they were different between patients with schizophrenia and the control in the previous studies. According to these studies, we can speculate that football habits might improve such set-shifting function and spatial working memory, which warrant further investigation with a larger sample size. Note that ROCFT requires a finer and longer time as a visual working memory task than CANTAB SWM, and TMT B requires more complex executive functioning using the numbers and alphabet character, compared with CANTAB IED using only simple figures. In RTI simple and five-choice reaction and movement time, there were fewer previous studies with inconsistent results in the patients [41, 42]. Our RTI results suggest that the reaction and movement times of the patients were impaired in the more complex tasks than the simplified ones. The ROCFT immediate reproduction score, indicating the sensory-perceptual ability, also demonstrated the inconsistent results between the previous studies [43, 44].

In the correlation and partial correlation analysis, the negative partial correlation between the pass angle and EDS errors in CANTAB IED (both of which did not indicate significant differences between the groups), suggests that set-shifting function may be related to the behavior in the 3-vs-1 task. We found the significant relationship only in the patients with schizophrenia, not in healthy controls, suggesting that the factors behind the relationship might be more complicated in the control group. The positive partial correlation between the angular displacement and TMT B, both of which showed significant differences between the groups. It suggests that the patients with impaired executive functioning may increase the displacement in the pass angle (which was also an impaired ability). It might be explained by the compensatory adaptation to the 3-vs-1 task which may predictively increase the displacement possibly as a result of the long-term training.

There are several recommendations for future studies. The first is the increase in the number of patients with schizophrenia and the controls. Since patients with schizophrenia may have heterogeneity, the characteristics of the small number of participants in our study may not be consistent with those in previous studies. Moreover, if the number of participants is increased, we can expect to obtain the evidence that the patients can improve the cognitive and interpersonal coordination performance to a similar level to healthy controls. The second is more hypothesis-driven studies based on the results of our study. For example, the studies using social cognitive tasks such as biological motion perception [13] and those comparing participants with and without sports habits should be further investigated. The third is to investigate the effect of neurological soft signs [22] in patients with schizophrenia on their motor coordination performances. The fourth is the effect of laterality (i.e., footedness) on the performance [45], whereas our tasks may have a smaller effect of the footedness on the performance than the above agility task. The last is the longitudinal study such as using team sports training. It would be warranted to examine the effects of team sports habits on interpersonal coordination and visual cognitions such as investigated in this study.

## Supporting information

S1 Data Analyzed data. (XLSX)
